# Initiation of Type 2 Diabetes Mellitus Medications in the NGHA Healthcare System in Saudi Arabia: Contemporary Trends

**DOI:** 10.1016/j.curtheres.2025.100809

**Published:** 2025-07-23

**Authors:** Mesnad S. Alyabsi, Lolwah Almousa, Anwar H. Alqarni, Asma Aldawsari, Lubna Alnasser, Adel F. Almutairi

**Affiliations:** a Department of Population Health, King Abdullah International Medical Research Center, Riyadh, Saudi Arabia; b Department of Population Health, King Saud bin Abdulaziz University for Health Sciences, Riyadh, Saudi Arabia; c Health and Rehabilitation Sciences College, Princess Nourah Bint Abdulrahman University, Riyadh, Saudi Arabia; d Department of Research Quality Management, King Abdullah International Medical Research Center, Riyadh, Saudi Arabia

**Keywords:** Antidiabetics, Diabetes Mellitus, Pharmacoepidemiology, Saudi Arabia

## Abstract

**Background:**

Continuous updates in management guidelines for type 2 diabetes mellitus (T2DM) have affected treatment patterns. In this study, we aimed to identify the prevalence of initiating various antidiabetic medications and analyze differences in patient characteristics based on the type of treatment initiated.

**Methods:**

This cross-sectional study used data retrieved from the electronic medical records of the National Guard Health Affairs for all patients diagnosed with T2DM who initiated any antidiabetic therapy between January 2018 and May 2022. Patient data were presented using frequencies, percentages, means, and standard deviations where applicable. The antidiabetic classes investigated include metformin, insulin, sulfonylureas, thiazolidinediones, dipeptidyl peptidase-4 inhibitors, glucagon-like peptide-1 receptor agonists, and sodium–glucose cotransporter-2 inhibitors.

**Results:**

In total, 433 patients with T2DM were included in this study, most of whom were females (55.66% vs 44.34%) and obese (61.20%), with a mean age of 53.29 years (SD ± 15.22). Monotherapy was the most commonly initiated approach (66.28%), with insulin being the most prescribed monotherapy (29.10%). The most frequent combination therapy was metformin and sulfonylureas (7.16%). Overall, the most initiated medication was metformin, accounting for 37.12% of all prescriptions. Additionally, there was an increasing trend in prescribing newer medications, such as GLP-1 receptor agonists (8.70%) and SGLT-2 inhibitors (11.11%), for newly diagnosed patients in 2021.

**Conclusion:**

The initiation of novel antidiabetic medications has increased over the study period, reflecting recent updates in T2DM management guidelines. However, further understanding of their benefits is required for optimal patient care.

## Introduction

Type 2 diabetes mellitus (T2DM) remains a significant global health concern, affecting 6.28% of the world’s population.^1^ This prevalence is expected to double, reaching 12.2% by 2045.^2^ In Saudi Arabia, which ranks seventh globally for diabetes prevalence, approximately 16.4% of the population has diabetes, with nearly 3 million individuals in the prediabetes stage.^3^^,^^4^

If left untreated, T2DM can lead to severe complications, including cardiovascular, ophthalmological, nephrological, neurological, and other microvascular disorders.^5^ To address this, treatment strategies integrating lifestyle modifications with pharmacological interventions have been implemented.^6^ Since the introduction of sulfonylureas in the 1950s as the first antidiabetic agents,^7^ advancements in pharmacotherapy have led to the development of diverse antidiabetic classes and increasingly sophisticated treatment options.^8^ Antidiabetic agents are classified based on their pharmacodynamic effects on blood glucose regulation. Major classes of these agents include metformin, insulin, sulfonylureas, dipeptidyl peptidase-4 (DPP-4) inhibitors, glucagon-like peptide-1 (GLP-1) receptor agonists, thiazolidinediones (TZDs), and sodium–glucose cotransporter-2 (SGLT2) inhibitors.^9^

Metformin remains the cornerstone of initial T2DM management, as recommended by the American Diabetes Association.^8^ However, newer agents, such as SGLT2 inhibitors and GLP-1 receptor agonists, have gained recognition for their benefits beyond glycemic control, including reducing cardiovascular risk and slowing the progression of renal impairment.^10^ In addition, DPP-4 inhibitors offer weight loss benefits and cardiovascular protection for high-risk patients.^11^

Recently, there has been a notable shift in the initiation patterns of antidiabetic medications. Updates in the standard of care, incorporating advancements in antidiabetic agents, have influenced first-line T2DM management patterns.^12^ A recent study highlighted a steady increase in the prescription of SGLT2 inhibitors and GLP-1 receptor agonists, particularly among high-risk patients.^13^ Conversely, although sulfonylurea (SU) is known for its affordability, its usage has declined significantly over the previous decades (dropping from 39.9% to 24.5%) due to reported associations with cardiovascular events, weight gain, and hypoglycemia.^14^^,^^15^

Previous studies have investigated the trends in antidiabetic medications use in patients with T2DM in Saudi Arabia. However, some of these studies lack the inclusion of Insulin or did not consider the prescription of different types of antidiabetic combinations. This study aims to provide a comprehensive insight into the evolving landscape of T2DM management using real-world setting. Therefore, we sought to identify the prevalence of initiating antidiabetic agents, and to describe the differences in patient characteristics based on the type of the initiated treatment.

## Methodology

### Data sources

This observational, cross-sectional study used the electronic medical record system of the National Guard Health Affairs (NGHA) linked to the diabetes mellitus (DM) registry. These records include data on all patients with DM diagnosed and treated at NGHA facilities across five regions of Saudi Arabia: Riyadh, Jeddah, Madinah, Al Ahsa, and Dammam. Patient data were meged using a unique identifier to allow for the integration of both clinical and demographic data. This study was approved by the Institutional Review Board of the King Abdullah International Medical Research Center (protocol number: NRR24/046/12). The deidentified datasets used in this study are not publicly available due to patient confidentiality concerns, but it can be obtained from the corresponding author upon reasonable request.

### Study population

The study included all patients diagnosed with T2DM who were 18 years or older and had initiated antidiabetic medications with at least one recorded prescription in the NGHA’s electronic medical record system between January 2018 and May 2022.

### Study variables

#### Patient characteristics

Demographic data extracted from the hospital records and DM registry included age at first prescription, sex, and body mass index (BMI). Clinical variables included HbA1c levels, fasting blood sugar levels, and diagnoses of cardiovascular or renal diseases at treatment initiation. The investigated medications were recommended by T2DM management guidelines, including metformin, insulin, sulfonylureas, TZDs, DPP-4 inhibitors, GLP-1 receptor agonists, and SGLT2 inhibitors.

### Outcome variables

The primary outcome of this study was the proportion of patients with T2DM who were newly initiated antidiabetic medications, including monotherapies and combination therapies, from January 2018 to May 2022.

### Statistical analysis

Descriptive statistics, including frequency, percentages, means, and standard deviations, were used to summarize the data. All data were cleaned, coded, and analyzed using SAS statistical software, Version 9.4 (SAS Institute Inc., Cary, NC).

### Results

In total, 433 patients with T2DM who initiated antidiabetic medications between 2018 and 2022 were included in this study. Their demographic and clinical characteristics are presented in Table 1. The mean age of the study population was 53.29 years (SD ± 15.22), with fluctuations over the study period, ranging from 54.26 years in 2018 to 46.14 years in 2022. The majority of the study population were females (55.66% vs 44.34%), had a mean BMI of 32.87 (SD ± 7.63), with obesity affecting 61.20% of the patients. Average HbA1c and fasting plasma glucose (FPG) levels were constant over the study years (HbA1c: 8.71 ± 2.47, FPG: 10.42 ± 5.45).

**Table 1:** Characteristics of patients with type 2 diabetes mellitus who initiated antidiabetic medications (2018–2022).

	All (N = 433)	2018 (N = 138)	2019 (N = 121)	2020 (N = 81)	2021 (N = 79)	2022 (Jan–May) (N = 14)
**Gender (%)**						
Female	241 (55.66%)	76 (55.07%)	66 (54.55%)	44 (54.32%)	47 (59.49%)	8 (57.14%)
Male	192 (44.34%)	62 (44.93%)	55 (45.45%)	37 (45.68%)	31 (39.24)	6 (42.86%)
**Age (Mean ± SD)**						
	53.29 ± 15.22	54.26 ± 14.79	53.76 ± 15.18	51.71 ± 15.45	53.74 ± 15.13	46.14 ± 18.60
**BMI (Mean ± SD)**						
	32.87 ± 7.63	33.83 ± 7.77	33.92 ± 8.36	31.15 ± 6.69	31.35 ± 6.52	32.93 ± 8.54
**Obesity (BMI > 30 kg/m^2^) (%)**						
	265 (61.20%)	94 (68.12%)	81 (66.94%)	42 (51.85%)	43 (54.43 %)	5 (35.71%)
**HbA1c (Mean ± SD)**						
	8.71 ± 2.47	8.90 ± 2.45	8.50 ± 2.59	7.97 ± 1.63	8.46 ± 2.17	10.95 ± 2.44
**FPG (Mean ± SD)**						
	10.42 ± 5.45	9.43 ± 4.00	9.73 ± 4.77	8.49 ± 2.64	8.16 ± 4.30	14.98 ± 6.28

BMI = body mass index; HbA1c = glycated hemoglobin; FPG = fasting plasma glucose.

Table 2 shows the characteristics of patients who initiated antidiabetic medication by the type of medication. The prescription patterns varied across medications and patient groups. TZDs and SGLT2 inhibitors were more commonly prescribed to males (80% and 60%, respectively), while the other medications showed relatively similar prescription patterns between the groups. The highest-initiated medication among patients with obesity was GLP-1 receptor agonists (81.08%), followed by metformin (63.36%) and DPP-4 inhibitors (59.42%). SGLT2 inhibitors were initiated in 20% of patients suffering from cardiovascular diseases but only in 6.67% of those with preexisting kidney-related diseases. For patients with renal disease, TZDs were the most frequently initiated antidiabetic medication, accounting for 20% of all prescriptions.

**Table 2:** Characteristics of patients who initiated antidiabetic treatment by the type of treatment (2018–2022).

	Metformin (N = 232)	Insulin (N = 184)	Sulfonylureas (N = 83)	DPP (N = 69)	GLP-1 receptor agonists (N = 37)	TZDs (N = 5)	SGLT2 (N = 15)
**Gender (%)**							
Female	140 (60.34%)	98 (53.26%)	51 (61.45%)	39 (56.52%)	19 (51.35%)	1 (20.00%)	6 (40.00%)
Male	92 (39.66%)	86 (46.74%)	32 (38.55%)	30 (43.48%)	18 (48.65%)	4 (80.00%)	9 (60.00%)
**Age**							
	52.89 ± 14.67	52.20 ± 16.23	55.62 ± 13.83	58.11 ± 14.33	54.89 ± 9.86	58.8 ± 5.63	56.93 ± 12.54
**BMI (Mean ± SD)**							
	33.33 ± 7.63	32.27 ± 8.22	33.16 ± 8.15	31.72 ± 6.55	36.30 ± 8.05	30.32 ± 2.48	31.14 ± 6.29
**Obesity (BMI > 30 kg/m^2^) (%)**							
	147 (63.36%)	106 (57.61%)	49 (59.04%)	41 (59.42%)	30 (81.08%)	2 (40.00%)	6 (40.00%)
**HbA1c (Mean ± SD)**							
	8.50 ± 2.39	-	9.77 ± 2.30	8.81 ± 2.03	-	8.05 ± 1.34	9.62 ± 2.76
**FPG (Mean ± SD)**							
	-	12.56 ± 6.65	11.56 ± 4.98	9.78 ± 3.98	9.37 ± 4.49	8.63 ± 2.71	8.5 ±.62
**Cardiovascular disease (%)**							
Yes	14 (6.03%)	12 (6.52%)	7 (8.43%)	4 (5.80%)	6 (16.22%)	-	3 (20.00%)
No	218 (93.97%)	172 (93.48%)	76 (91.57%)	65 (94.20%)	31 (83.78%)	5 (100%)	12 (80.00%)
**Renal disease (%)**							
Yes	8 (3.45%)	12 (6.52%)	5 (6.02%)	3 (4.35%)	3 (8.11%)	1 (20.00%)	1 (6.67%)
No	224 (96.55%)	172 (93.48%)	78 (93.98%)	66 (95.65%)	34 (91.89%)	4 (80.00%)	14 (93.33%)

BMI = body mass index; HbA1c = glycated hemoglobin; FPG = fasting plasma glucose.

In addition, Table 3 demonstrates the first-line treatments, stratified by initiation year and type. Monotherapy was the most common treatment approach throughout the study period (66.28%), with insulin comprising 29.10% of monotherapies. Metformin was also extensively used, serving as the top-prescribed monotherapy in 2018 and 2019, covering approximately 30% of the studied cohort during those years. Dual therapy increased significantly, comprising 23.91% of prescriptions in 2018 and reaching 50% by 2022. The most prescribed combination therapy was metformin and sulfonylureas, accounting for 7.16% of prescriptions. Trio and quadruple therapies were rarely used, with no quadruple therapy prescriptions recorded from 2020 onward.

**Table 3:** Trends in the initiated medications for type 2 diabetes mellitus by therapy class (2018–2022).

Medication	All (N = 433)	2018 (N = 138)	2019 (N = 121)	2020 (N = 81)	2021 (N = 79)	2022 (Jan–May) (N = 14)
**Monotherapy (%)**	**287 (66.28%)**	**88 (63.77%)**	**81 (66.94%)**	**58 (71.60%)**	**54 (68.35%)**	**6 (42.86%)**
Metformin	109 (25.17%)	41 (29.71%)	34 (28.10%)	18 (22.22%)	13 (16.46%)	3 (21.43%)
Insulin	126 (29.10%)	35 (25.36%)	32 (26.45%)	31 (38.27%)	25 (31.65%)	3 (21.43%)
Sulfonylureas	15 (3.46%)	8 (5.80%)	6 (4.96%)	-	1 (1.27%)	-
DPP	13 (3.00%)	4 (2.90%)	2 (1.65%)	3 (3.70%)	4 (5.06%)	-
GLP-1 receptor agonists	17 (3.92%)	-	6 (4.96%)	6 (7.41%)	5 (6.33%)	-
TZDs	1 (0.23%)	-	1 (0.83%)	-	-	-
SGLT2	6 (1.38%)	-	-	-	6 (7.59%)	-
**Dual therapy (%)**	**105 (24.25%)**	**33 (23.91%)**	**28 (23.14%)**	**16 (13.58%)**	**21 (26.58%)**	**7 (50.00%)**
**Metformin+**						
Insulin	25 (5.77%)	10 (7.25%)	7 (5.79%)	5 (6.17%)	2 (2.53%)	1 (7.14%)
Sulfonylureas	31 (7.16%)	12 (8.70%)	9 (7.44%)	3 (3.70%)	4 (5.06%)	3 (21.43%)
DPP	21 (4.85%)	8 (5.80%)	3 (2.48%)	3 (3.70%)	6 (7.59%)	1 (7.14%)
GLP-1 agonists	5 (1.15%)	1 (0.72%)	2 (1.65%)	-	2 (2.53%)	-
SGLT2	2 (0.46%)	-	-	-	1 (1.27%)	1 (7.14%)
**Insulin+**						
DPP	2 (0.46%)	-	1 (0.83%)	1 (1.23%)	-	-
GLP-1 agonists	4 (0.92%)	1 (0.72%)	2 (1.65%)	1 (1.23%)	-	-
SGLT2	3 (0.69%)	-	-	1 (1.23%)	2 (2.53%)	-
Sulfonylureas	2 (0.46%)	-	2 (1.65%)	-	-	-
**DPP+**						
Sulfonylureas	5 (1.15%)	1 (0.72%)	2 (1.65%)	2 (2.47%)	-	-
**GLP-1 agonists +**						
Sulfonylureas	2 (0.46%)	-	-	-	1 (1.27%)	1 (7.14%)
SGLT2	2 (0.46%)	-	-	-	2 (2.53%)	-
**SGLT2+**						
TZDs	1 (0.23%)	-	-	-	1 (1.27%)	-
**Trio therapy (%)**	**36 (8.31%)**	**13 (9.42%)**	**11 (9.09%)**	**7 (8.64%)**	**4 (5.06%)**	**1 (7.14%)**
**Metformin+ DPP+**						
Insulin	9 (2.08%)	4 (2.90%)	2 (1.65%)	2 (2.47%)	1 (1.27%)	-
Sulfonylureas	15 (3.46%)	4 (2.90%)	6 (4.96%)	3 (3.70%)	2 (2.53%)	-
**Metformin+ Insulin+**						
Sulfonylureas	5 (1.15%)	3 (2.17%)	2 (1.65%)	-	-	-
Other	7 (1.62%)	2 (1.45%)	1 (0.83%)	2 (2.47%)	1 (1.27%)	1 (7.14%)
**Quadruple Therapy (%)**	**5 (1.15%)**	**4 (2.90%)**	**1 (0.83%)**	**0 (0.00%)**	**0 (0.00%)**	**0 (0.00%)**
S M I D	2 (0.46%)	1 (0.72%)	1 (0.83%)	-	-	-
Other	3 (0.69%)	3 (2.17%)	-	-	-	-

S = sulfonylureas; M = metformin; I = insulin; D = dipeptidyl peptidase-4 inhibitors.

**Figure 1:**
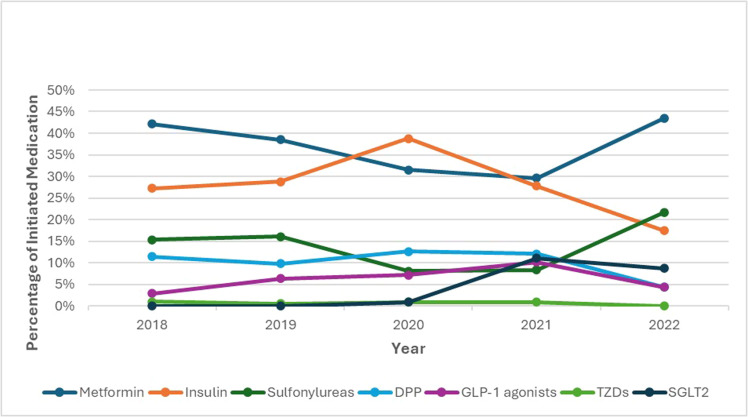
Trends in the initiated medications for type 2 diabetes mellitus (2018–2022).

Figure 1 shows the trends in the initiated medications for T2DM management from 2018 to 2022. The prescription rate of insulin has increased from 27.27% in 2018 to 38.74% in 2020 before dropping to 17.39% in 2022. Sulfonylureas prescriptions decreased from 15.31% in 2018 to 8.11% in 2020 but rebounded to 21.74% in 2022. TZDs were the least prescribed drugs, with a relatively stable prescription rate throughout the study period, accounting for about 0.90% of prescriptions. SGLT2 inhibitors were introduced to NGHA patients in 2020, with their usage increasing to 11.11% and 8.70% by 2021 and 2022, respectively.

### Discussion

In this study, we aimed to investigate contemporary trends in the initiation of antidiabetic medications among the Saudi population. Most of the studied patients commenced treatment with monotherapy. Insulin was the most frequently prescribed monotherapy for T2DM, peaking at 38.74% in 2020. This was followed by metformin, which was the predominant monotherapy in 2018 and 2019. In addition, a gradual shift toward prescribing newer antidiabetic medications was observed, reflecting the global trend toward more innovative and effective treatments beyond merely controlling blood glucose levels.

While insulin was the most prescribed monotherapy, metformin remained the most commonly initiated antidiabetic overall (37.12%). Current guidelines recommend metformin for individuals newly diagnosed with T2DM due to its proven safety, efficacy in regulating blood glucose levels, weight benefits, and low risk of hypoglycemia.^16^ This trend aligns with findings from previous studies.^12^^,^^13^^,^^17^

Alternative medications are available for patients for whom metformin is contraindicated, such as those with renal disease. Although GLP-1 receptor agonists and SGLT2 inhibitors offer benefits for patients with renal disease, our results revealed a relatively low initiation rate for these medications, consistent with a previous study.^13^ The relatively recent approval of GLP-1 receptor agonists in 2005 and SGLT2 inhibitors in 2013 may explain their lower usage compared with the over 60 years of clinical experience with metformin and sulfonylureas.^18^ We observed an increasing trend in the use of DPP-4 inhibitors as monotherapy. However, their overall utilization remained stable throughout the study period, contrary to a previous study, which reported a significant increase in the initiation of DPP-4 inhibitors, making them the third most commonly prescribed first-line treatment.^19^ Although the definition of antidiabetic initiation may vary across studies, the trends observed in our study partially align with those observed in other countries,^12^^,^^20^ while differing in some respects.^21^

We also investigated the characteristics of patients with T2DM based on the treatment initiated. Despite recommendations to avoid prescribing SU to patients with obesity,^22^ over half of the SU prescriptions in our study were given to these patients (59.04%). SU is commonly associated with weight gain, primarily due to its mechanism of action, which stimulates increased appetite to prevent hypoglycemia.^23^ The initiation of GLP-1 receptor agonists has notably increased from 2.87% in 2018 to 10.19% in 2021. This increase is largely due to the recent approval of the drug for weight reduction, expanding its use in managing chronic obesity.^24^ GLP-1 receptor agonists and SGLT-2 inhibitors were initiated mostly in patients with cardiovascular or renal comorbidities. This trend is supported by clinical trials involving patients with T2DM that show the beneficial effects of these medications beyond glycemic control, including slowing the progression of kidney disease and reducing cardiovascular events,^25^, ^26^, ^27^ which was also shown in another study on their overall utilization.^28^

Patients who started insulin had a mean FPG level of 12.56 ± 6.65 mmol/L, reflecting the clinical consensus that insulin is often prescribed for patients with T2DM when their blood glucose levels are elevated.^29^ Early initiation of insulin in patients with hyperglycemia has been shown to reduce glucotoxicity, preserve β-cell function, and minimize the development of chronic complications.^30^

This study had several limitations. First, we focused only on the trends in initiating antidiabetic treatments and could not track longitudinal factors, such as medication switches or add-ons, over time, which may provide a comprehensive understanding of current trends. Second, due to the nature of the data source, the quality and completeness of the medical records may have been compromised by missing or inaccurate information, particularly regarding HbA1c and FPG levels. In addition, physician preferences and familiarity with specific medications may have influenced medication choices and initiation trends. To the best of our knowledge, this is the first study conducted in Saudi Arabia to comprehensively describe trends in antidiabetic medication initiation using real-world patient data. This approach provides a comprehensive reflection of actual clinical practices and aligns with evolving clinical guidelines. However, the results are restricted to NGHA patients and may not be generalizable to other healthcare settings or populations. Given these strengths and limitations, future research should incorporate detailed information on the overall utilization of antidiabetics, medication adherence, and physician decision-making over a longer timeframe. Furthermore, examining the relationship between these factors and the type of treatment initiated would be valuable.

In conclusion, despite the fact that insulin was the top prescribed monotherapy, metformin emerged as the most initiated medication among all types of antidiabetic prescriptions for managing T2DM in Saudi Arabia, aligning with international and national treatment guidelines. There is a growing trend in prescribing newer medications, such as GLP-1 receptor agonists and SGLT-2 inhibitors, for newly diagnosed patients. However, the adoption of alternative treatment options must be encouraged to keep pace with recent updates, highlighting the need for greater awareness of their advantages to ensure optimal patient care.

## Author's Contribution

Mesnad Alyabsi: Conceptualization, Methodology, Writing. Lolwa Almousa: Data curation, data analysis, writing. Anwar alqarni: Data analysis, writing. Asma Aldawsari: Data curation, writing. Lubna Alnasser: Data access, writing, supervision. Adel Almutairi: Supervision, software, writing.

## Declaration of competing interest

The authors declare that they have no known competing financial interests or personal relationships that could have appeared to influence the work reported in this paper.

## Appendix Supplementary materials

Image, application 1
